# New activities for the anti-tumor agent trabectedin: taking two birds with one stone

**DOI:** 10.18632/oncotarget.968

**Published:** 2013-04-08

**Authors:** Maurizio D'Incalci, Roberta Frapolli, Giovanni Germano, Paola Allavena

**Affiliations:** Mario Negri Institute for Pharmacological Research, Milan, Italy; Mario Negri Institute for Pharmacological Research, Milan, Italy; Clinical and Research Institute Humanitas, Rozzano, Milan, Italy; Clinical and Research Institute Humanitas, Rozzano, Milan, Italy

In the last decade the neoplastic cell-centric paradigm is now changing in a stroma-centric paradigm, focusing on the tumor micro-environment. This is populated by inflammatory leukocytes, activated fibroblasts and newly formed vessels, which influence each other in a complex and dynamic cross-talk with neoplastic cells. It is now established that in most cancers Tumor-Associated Macrophages (TAM) display pro-tumor functions (e.g. increase tumor cell proliferation and survival, aid tumor cell dissemination, promote angiogenesis and matrix remodelling) [1-3]. Particular emphasis in cancer therapy has been put in combination strategies to attack both tumor cells and the stroma. We have recently reported in a paper published in Cancer Cell in February 2013 that the anti-tumor agent trabectedin has important modulatory effects on the tumor micro-environment [4].

Trabectedin is an anti-tumor compound registered in Europe and in several other countries, for the second line treatment of soft tissue sarcoma (STS) and for ovarian cancer in combination with liposomal doxorubicin [5-7]. This compound was originally extracted from a marine organism, the Tunicate Ecteinascidia, and is now synthetically produced by the Spanish company PharmaMar. Trabectedin is a minor groove DNA binder able to efficiently block cancer cell proliferation. However, this is only part of its several mechanisms of action; further studies, revealed inhibition on the activity of selected transcription factors, which translate – among other effects – in the reduced production of several inflammatory mediators like the chemokines CCL2 and CXCL8, the cytokine IL-6 and the angiogenic factor VEGF [8].

An effect on the micro-environment was suspected by the clinical evidence of atypical patterns of response: for instance, in patients with liposarcoma, a decrease in tumor density may occur without tumor shrinkage for several courses, eventually resulting in size decrease. In other patients, clinical responses take place only after several cycles of therapy. Indeed, STS patients have less than 10% responses (by RECIST criteria) but up to 50% stable disease and long overall survival [6-7]. A particularly high susceptibility to trabectedin has been observed in translocated-related soft tissue sarcoma. In myxoid liposarcoma, whose pathogenesis is related to a balanced translocation most commonly t(12;16)(q13;q12) fusing the FUS gene with the CHOP gene, trabectedin induces a prolonged response with a decrease in the vascular network and adipocytic differentiation in a large fraction of patients [7]. The FUS-CHOP chimera alters the transcription of several genes including some encoding for proteins involved in pro-inflammatory and angiogenic networks and in the repression of adipocytic maturation; indeed, the exquisite antitumor activity of trabectedin seems to be related to its ability to inhibit the aberrant transcription factor FUS-CHOP [5].

**Figure 1:**
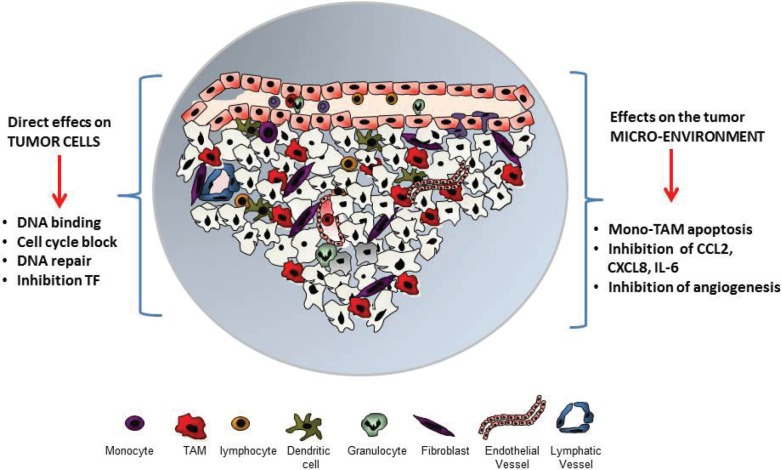
The anti-tumor agent trabectedin has direct effects on tumor cells, blocking cell cycle and proliferation It also interferes with DNA repair mechanisms and with the activity of selected transcription factors (TF). At the level of the tumor micro-environment trabectedin is cytotoxic to monocytes and Tumor-Associated Macrophages (TAM); inhibits angiogenesis and the production of several inflammatory mediators.

In the Cancer Cell paper [4] we have used trabectedin in different mouse tumor models and investigated its effects on the tumor micro-environment, more specifically on leukocytes, angiogenesis, and on the expression of inflammatory mediators. We found that in treated mice trabectedin significantly decreased the number of blood monocytes and of tumor macrophages, having direct cytotoxic effects on this lineage, but not on other leukocyte subsets, such as neutrophils and lymphocytes (Fig. 1). We further demonstrated that treated tumors had a reduced vessel network. Similar results were found in human STS samples from patients receiving trabectedin as neo-adjuvant therapy: a marked decrease in blood vessels and in the tumor infiltrating macrophages. Using human liposarcoma xenografts grown in nude mice, we observed also a significant decrease in the expression of relevant mediators such as CCL2 and CXCL8 [8].

Overall, this compound has two major targets: it directly inhibits the neoplastic compartment but also affects the tumor micro-environment, in particular the macrophages and their pro-tumoral functions.

Trabectedin is currently used to treat a limited number of tumor types, and as second line therapy. Our findings that trabectedin has wider mechanism of action and strikes the whole micro-environment may stimulate its use in less advanced neoplastic settings and in tumors of different histology.
